# Predicting readmission of heart failure patients using automated follow-up calls

**DOI:** 10.1186/s12911-015-0144-8

**Published:** 2015-03-29

**Authors:** Shelby Inouye, Vasileios Bouras, Eric Shouldis, Adam Johnstone, Zachary Silverzweig, Pallav Kosuri

**Affiliations:** Keck School of Medicine of USC, Los Angeles, CA USA; CipherHealth, New York, NY USA; Department of Internal Medicine, Charleston Area Medical Center, Charleston, WV USA; Department of Chemistry and Chemical Biology, Harvard University, 12 Oxford Street, Cambridge, MA 02138 USA

**Keywords:** Heart failure, Risk assessment, Post-discharge follow-up, Readmission prediction

## Abstract

**Background:**

Readmission rates for patients with heart failure (HF) remain high. Many efforts to identify patients at high risk for readmission focus on patient demographics or on measures taken in the hospital. We evaluated a method for risk assessment that depends on patient self-report following discharge from the hospital.

**Methods:**

In this study, we investigated whether automated calls could be used to identify patients who are at a higher risk of readmission within 30 days. An automated multi-call follow-up program was deployed with 1095 discharged HF patients. During each call, the patient reported his or her general health status. Patients were grouped by the trend of their responses over the two calls, and their unadjusted 30-day readmission rates were compared. Pearson’s chi-square test was used to evaluate whether readmission risk was independent of response trend.

**Results:**

Of the 1095 patients participating in the program, 837 (76%) responded to the general status question in at least one of the calls and 515 (47%) patients responded to the general status question in both calls. Out of the 89 patients exhibiting a negative response trend, 37% were readmitted. By contrast, the 97 patients showing a positive trend and the 329 patients showing a neutral trend were readmitted at rates of 16% and 14% respectively. The dependence of readmission on trend group was statistically significant (P < 0.0001).

**Conclusions:**

Patients at an elevated risk of readmission can be identified based on the trend of their responses to automated follow-up calls. This presents a simple method for risk stratification based on patient self-assessment.

## Background

An estimated 5.1 million people in the United States suffer from heart failure, and approximately 550,000 new diagnoses are made each year [1,2]. Although notable improvements have been made in the treatment of patients diagnosed with heart failure (HF), the national average readmission rate remains stagnant, with approximately one in four patients readmitted within 30 days of discharge [3]. In addition to the excessive trauma this may cause for the patient, readmissions can also place a large financial burden on the hospital. In FY2013 alone, Centers for Medicare and Medicaid Services penalized 2,200 U.S. hospitals a combined $280 million [4].

While it may not be possible to determine the exact proportion of preventable readmissions, evidence shows that comprehensive discharge planning and early follow-up can reduce the likelihood of readmission in HF patients [5,6]. The American Heart Association has advocated for post-discharge follow-up and has published a set of guidelines for post-discharge telephone calls [7]. However, due to the high volume of discharged patients, it is likely necessary to perform targeted interventions based on risk stratification. Prior studies have identified demographic and clinical patient data, such as marital status, insurance status, and comorbidities, as predictive factors for readmission [8-15]. While these models may provide considerable value, they tend to omit the potentially significant component of the patient’s self-reported general condition. One efficient method for obtaining patient information post-discharge is through the use of automated calling. Automated calls have been used in many studies to monitor patients and attempt to minimize readmission [16,17]. However, despite the often unique insight provided into a patient’s condition, patient response data have yet to be used as an effective means for risk stratification.

In this study, we investigated whether automated calls could be used to identify patients with HF who were at a higher risk of readmission within 30 days of hospital discharge. Our analysis showed that for this category of patients, self-assessment could provide a simple and efficient means for risk stratification.

## Methods

### Study population and eligibility

The study population was comprised of individuals enrolled in an automated post-discharge follow-up call program. The program was initiated at Charleston Area Medical Center (CAMC) in December of 2010 with the purpose of improving quality of care and patient outcomes. All enrolled patients for this study were discharged from CAMC in Charleston, West Virginia between December 2010 and September 2012. Individuals eligible for the call program were over 18 years of age, English-speaking, had a valid phone number, and had been admitted with a diagnosis of HF.

The automated call program was used to deliver information to CAMC clinicians regarding the patient’s condition following discharge. A third party executed CAMC's Business Association Agreement prior to providing any automated call services. The third party was in full compliance with all HIPAA standards, rules and regulations. The study was performed using data acquired from a program that was implemented for the purpose of improving care management services at CAMC. Therefore, there was no requirement for external institutional review board approval [18].

### Follow-up call design and protocol

The call script was generated via a collaborative effort between the third party (CipherHealth LLC, New York) and physicians at CAMC and JFK Medical Center in Edison, New Jersey. Follow-up questions were formulated based on best practice guidelines published by the American Heart Association, and the American College of Cardiology [7,19].

Upon discharge from the hospital, patient information was stored in the third party database. The call program consisted of two automated phone calls: patients received the first call within 48 hours of discharge and the second call seven days later. No calls were made on weekends, so if a patient was scheduled to be called during the weekend, his/her call was transferred to Monday. Therefore, patients who were discharged on Thursday or Friday, received their first call (two days after discharge) on Monday instead of Saturday or Sunday, respectively. Patients who were discharged during the weekend received their second call (nine days after discharge) on Monday after the following weekend instead of Saturday or Sunday respectively. Patients input their responses using a touch-tone phone. On the first call, four questions related to general health status, medications, follow-up appointments, and weight gain were asked. On the second call, the same inquiries were made, and a fifth question regarding maintenance of a low-sodium diet was included.

We hypothesized that responses to the general health status would provide predictive information about a patient’s readmission risk. The general status question reads, “How are you feeling compared to when you were discharged from the hospital?” Possible responses were 1-better, 2-about the same, 3-worse, or 4-much worse. A trend was generated based on patients’ responses to the general status question on the second call as compared to the first. Patients who responded more positively on the second call than on the first were considered to have a positive trend. Patients who responded more negatively on the second call than on the first were considered to have a negative trend. We hypothesized that patients showing a negative trend would be more likely to be readmitted than those with a positive or neutral trend.

Several measures were taken to maximize compliance. Patients were notified by the nursing staff of the approximate time and date of the calls. In addition, the automated calls were made using a phone number from the hospital, and the voice talent reflected the accent of the region. In the event of a missed call, the patient received a voicemail explaining that another attempt would be made in the near future. Up to four call attempts would be made on the scheduled call day.

### Data collection and statistical analysis

Patient response data were delivered via automatic reports. Prior to analysis, all identifying data such as name, date of birth, and medical record number were removed. For the trend analysis, patients were assigned a value of “positive”, “neutral”, or “negative” based on the trend of their answers. Readmissions within 30 days of discharge were recorded and unadjusted readmission rates were calculated for each trend group. Pearson’s chi-square test of independence was used to assess whether the unadjusted readmission rates were independent of response trend group.

## Results

**Figure 1:**
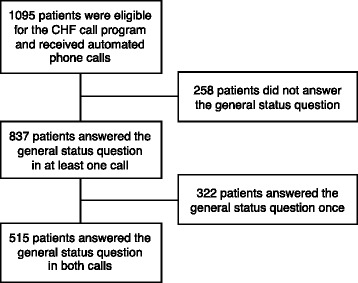
**Patient inclusion flow chart.**

Out of the 1095 HF patients selected for the study, 837 patients (76%) responded to the general status question in at least one call, and 515 patients (47%) responded to the general status question in both calls (Figure 1). A total of 244 patients (22%) were readmitted within 30 days of discharge from the hospital, which is consistent with the nationwide average rate of readmission [20]. The outcomes for different patient groups are summarized in Table 1. The rate of readmissions among patients who answered the general status question at least once was 21% as compared to 27% for those who did not answer the question. This difference was found to be statistically significant with *P* = 0.03.

**Table 1:** **Readmission rates by patient group**

	**Readmitted patients**	**Not readmitted patients**	**Total patients**	**Risk index**	***P-*** **value**
**All patients**	244	851	1095	22%	
**General status question**					
**Responded to general status question**					0.0323
Yes	174	663	837	21%	
No	70	188	258	27%	
Total	244	851	1095	22%	
**Response trend**					<0.0001
Neutral trend	46	283	329	14%	
Positive trend	16	81	97	16%	
Negative trend	33	56	89	37%	
Total	95	420	515	18%	
**First call response**					0.0324
Better	80	363	443	18%	
Same	60	164	224	27%	
Worse or much worse	14	49	63	22%	
Total	154	576	730	21%	
**Second call response**					<0.0001
Better	53	359	412	13%	
Same	37	115	152	24%	
Worse or much worse	25	33	58	43%	
Total	115	507	622	18%	

Of the 515 patients who completed both follow-up calls, 89 exhibited a negative response trend, 329 exhibited a neutral trend, and 97 exhibited a positive trend. Among patients with a negative trend, the readmission rate was 37%. Among patients with positive or neutral trends, the readmission rates were 16% and 14%, respectively. With a *P* value less than 0.0001, trend group was found to be a significant predictor of readmission rate.

Further analysis revealed a relationship between readmission probability and the patient’s self-assessed status in the second follow-up call (*P* < 0.0001). 622 patients answered the general health status question in the second call. Of those patients, 412 responded feeling better, 152 responded feeling the same, and 58 responded feeling worse or much worse. The readmission rates for patients feeling better, same, and worse/much worse were 13%, 24%, and 43%, respectively. Results from the first call also revealed a difference in readmission rate among the groups, however with less significance (*P* = 0.03).

## Discussion

### Predicting readmission

Our study found that patients who responded to two automated follow-up calls could be stratified by readmission risk based on their self-assessments of health in two automated phone calls. Patients who displayed a self-reported decline in condition following discharge were more than twice as likely to be readmitted as those who reported a neutral or improved condition over the two phone calls.

Useful risk information was also obtained from the second call alone. Patients who responded negatively were readmitted almost three times as frequently as patients who responded positively or neutrally. These two methods could be complementary, since these latter high-risk patients were not necessarily represented in the trend group analysis (these patients may not have answered the first call). Furthermore, these patients may have responded negatively on both calls and thus have been included in the neutral trend group.

This study takes a new approach to risk stratification. Many preceding efforts have been made to stratify patients based on medical records and data obtained during the hospital stay. For instance, Krumholz et al. reviewed 2,176 patients in 18 hospitals and derived a model comprising four independent predictors, which included hospitalization in the prior year, medical history of HF, medical history of diabetes mellitus, and serum creatinine levels at discharge [9]. Philbin and DiSalvo accessed a data set including 42,731 patients in 236 hospitals and derived a risk score based on 11 variables, including black race/ethnicity, primary insurance of Medicare or Medicaid, medical history of ischemic heart disease, the use of telemetry during hospitalization, etc [10]. Although successful within the scope of their studies, models such as these have been shown to lack consistency when compared to other studies. In a review of statistical models for predicting HF readmission, 117 studies were examined and it was discovered that few characteristics were consistently associated with readmission [8]. With regard to risk stratification, no studies to date have demonstrated strong model discrimination for readmission [14,21].

We speculate that a risk model that includes dynamic patient-reported data, such as the data recorded in this study, may help strengthen discrimination.

### Preventing readmission

With the objective of improving healthcare quality and reducing costs, the United States government has increasingly encouraged hospitals to reduce preventable readmissions. In 2009, Medicare began publicly reporting 30-day risk-standardized readmission rates for HF, acute myocardial infarction, and pneumonia [22]. Although quality of inpatient care is an important factor associated with early readmission, there is also evidence for the efficacy of reaching out to high-risk patients following discharge [16,23]. A randomized controlled study showed that patients with HF who received telephone care post discharge had 84% lower HF-related readmission charges (*P* < 0.05) than the usual care group. This result suggests that follow-up care may lead to reductions in readmissions, emergency visits, and cost of care [24]. More recently, a Cochrane review of 30 peer-reviewed randomized controlled trials found that telemonitoring and structured telephone support decreased the rate of hospitalization in patients with HF [16].

Studies have also presented contrary evidence. Following the Cochrane review, a 2012 review of studies involving remote monitoring of patients with HF showed inconsistent results with regard to outcome improvement [17]. Of importance, however, were the inclusion criteria for the studies analyzed in the referenced reviews. In the Cochrane review, a program was classified as being “structured telephone support” if the remote care were delivered using simply a telephone, and a program was considered “telemonitoring” if there were digital transmission of physiologic or other non-invasive data [16]. This is contrasted with the 2012 review in which data from more invasive means such as implanted devices were included [17]. It is possible that certain interventional studies reported high readmission rates due to increased anxiety or even increased complications in patients using self-monitoring devices. The mixed results may also reflect the dependence on quality of follow-up.

Two large trials were recently published showing no significant difference in readmission for patients with heart failure participating in a telemonitoring program as compared to the control group [25,26]. In a study by Chaudhry, telemonitoring was performed using a telephone-based interactive-response system. Information was collected from discharged heart failure patients regarding symptoms and weight, and responses were reviewed by clinicians. Patients who triggered variances in their responses received follow-up care. While it was found that telemonitoring plus clinician intervention did not significantly improve readmission rates, the authors of that study pointed out that other telemonitoring studies may have yielded positive results due to an especially motivated follow-up staff [25,27]. Based on the varied success of such studies, it appears that the specific type of intervention plays a significant role in successfully preventing readmission.

By identifying high-risk patients, the method presented in this study can be used to more efficiently direct resources for follow-up care. In addition, such targeting could be of value when comparing the efficacy of various follow-up strategies.

### Limitations

We observed a difference in readmission rates between patients who adhered to the call program and patients who did not. Readmission risk was higher for non-adherent patients, which raises a concern about behavioral differences between these two patient groups. For instance, it is possible that patients who complied with the program were more inclined to follow their discharge instructions and therefore had a lower likelihood of readmission. It is also possible, however, that some of the non-adherent patients experienced very early readmission. Since dates of readmission were unavailable, we were not able to investigate this further. Regardless, it may be useful to identify characteristics associated with non-adherent patients since they displayed a higher rate of readmission. Our data showed that non-adherent patients tended to be younger than the patients who responded to calls. This might indicate that older patients consider post-discharge instructions more seriously. On the other hand, we found that the readmission rate did not differ significantly between different age groups, indicating that age difference alone could not explain the higher readmission rate of non-adherent patients.

Another important limitation is the lack of response to the first call. Of the 1095 patients called, 365 (33%) patients either failed to answer the first call or did not respond to the general status question on the first call. Studies show that 32% of 30-day readmissions in heart failure patients occur within the first seven days post-discharge [28]. Since the first call went out within 48 hours of discharge and the second call followed seven days later, a potentially significant number of patients at risk for readmission could not be identified. However, 107 of the 365 who failed to respond to the question on the first call responded on the second call, bringing the total number of completely non-adherent patients down to 258. Addressing this limitation, it is likely that stronger predictability could be attained by increasing the overall compliance of the program. This could be achieved by providing better information to the patients or by decreasing the number of questions asked in the phone calls. It is important to note that no tangible incentives were offered to patients to complete the automated calls, so the adherence rate should be representative of the general patient population.

This study focused on predicting readmissions post-discharge, and so we did not consider other factors that might contribute to risk of readmission. A future study could incorporate demographic information, health histories, and other relevant characteristics of automated call respondents to investigate which factors contribute to readmission risk.

The approach of dynamic self-report may present a potential advantage over risk stratification based on demographic or hospital data alone. Since it captures those who report a decline in condition regardless of their demographic profiles and health histories, self-assessment may identify individuals who are overlooked with alternative strategies. Furthermore, the method presented here can with advantage be used in situations when a patient's health history and clinical information is unavailable or unreliable. Nevertheless, in most cases, an integrated approach that uses several sources of data would likely prove advantageous.

## Conclusions

Hospitals and patients both stand to benefit from efficient post-discharge care. In particular, readmission rates can potentially be lowered through targeted interventions. An important step towards this goal is finding methods that can reliably stratify patients according to their readmission risk. In this study, we have described a simple method, using self-assessment through automated phone calls, to identify patients with HF who are at a high risk of readmission. Future studies may lead to a more integrated approach, whereby patient history and demographic data are utilized to further improve the accuracy of risk stratification. We conclude that automated phone calls present an effective initial means for hospitals to identify and engage in targeted follow-up of high-risk HF patients.
